# Should we treat mild gestational diabetes? An Australian multicentre retrospective cohort study

**DOI:** 10.1007/s00592-025-02548-6

**Published:** 2025-06-20

**Authors:** Victoria L. Rudland, Emily Hibbert, Jeff Flack, Tang Wong, Vincent W. Wong, Mark McLean, Dharmintra Pasupathy, David Simmons, N. Wah Cheung

**Affiliations:** 1 Sydney Medical School, Faculty of Medicine and Health, The University of Sydney, Camperdown, Sydney, NSW 2050 Australia; 2 Nepean Hospital, Sydney, Australia; 3 Bankstown-Lidcombe Hospital, Sydney, Australia; 4 Department of Medicine, UNSW, Sydney, Australia; 5 School of Medicine, Western Sydney University, Sydney, Australia; 6 Liverpool Hospital, Sydney, Australia; 7 Blacktown Hospital, Sydney, Australia; 8 Campbelltown Hospital, Sydney, Australia; 9 Westmead Hospital, Sydney, Australia

**Keywords:** Diabetes, Gestational, Glucose tolerance test, Pregnancy outcome, Pregnancy, Mild gestational diabetes

## Abstract

**Aims:**

The International Association of Diabetes in Pregnancy Study Groups (IADPSG) diagnostic criteria for gestational diabetes (GDM) were widely implemented in Australia, despite limited evidence of better pregnancy outcomes compared to the Australasian Diabetes in Pregnancy Society 1998 (ADIPS1998) criteria. We aimed to evaluate the effect of treatment on pregnancy outcomes for women with ‘mild’ GDM, defined as GDM diagnosed by one, but not both, sets of criteria.

**Methods:**

This multicentre, retrospective cohort study included 17,512 pregnant women in six neighbouring tertiary hospitals in Sydney, Australia, during 2016–2017, all of whom were screened for GDM using a three-point 75 g oral glucose tolerance test. Three hospitals diagnosed and treated GDM according to ADIPS1998 criteria, and three according to IADPSG criteria. For women with ‘mild’ GDM, we evaluated the effect of treatment versus no treatment on pregnancy outcomes. The primary outcome was large for gestational age. Secondary outcomes were small for gestational age, induction of labour, caesarean section, gestational hypertension, and preeclampsia.

**Results:**

2320 (13.2%) pregnant women had ‘mild’ GDM. Treatment of women with IADPSG-only GDM (i.e. fasting glucose 5.1–5.4 mmol/L (91–97 mg/dL) and/or 1-hour glucose ≥ 10.0 mmol/L (≥ 180 mg/dL)) was associated with less large for gestational age infants than no treatment (RR 0.66, 95%CI 0.49–0.88, *p* = 0.004) but more induction of labour (RR 1.55, 95%CI 1.03–2.34, *p* = 0.032). Treatment of women with ADIPS1998-only GDM (i.e. 2-hour glucose 8.0-8.4 mmol/L (144–151 mg/dL)) did not significantly change pregnancy outcomes compared with no treatment.

**Conclusions:**

This study highlights the importance of treating even mild IADPSG-GDM to improve pregnancy outcomes.

## Introduction

Glycaemic thresholds for gestational diabetes (GDM) diagnosis have created substantial national and international debate. In Australia, consensus criteria for GDM diagnosis using a 75 g oral glucose tolerance test (OGTT) were initially developed by a working party in 1991 [1] and formally adopted by the Australasian Diabetes in Pregnancy Society (ADIPS) in 1998 (fasting glucose ≥ 5.5 mmol/L (≥ 99 mg/dL) and/or 2-hour ≥ 8.0 mmol/L (≥ 144 mg/dL)) [2]. In 2008, the observational Hyperglycemia and Adverse Pregnancy Outcomes (HAPO) study demonstrated a linear relationship between glucose levels and adverse pregnancy outcomes on an antenatal OGTT at 24–32 weeks’ gestation [3]. With no clear threshold for increased risk, the International Association of Diabetes and Pregnancy Study Group (IADPSG) developed a consensus determination recommending diagnostic thresholds for GDM (fasting glucose ≥ 5.1 mmol/L (≥ 91 mg/dL), and/or 1-hour ≥ 10.0 mmol/L (≥ 180 mg/dL), and/or 2-hour ≥ 8.5 mmol/L (≥ 153 mg/dL)), based on a HAPO odds ratio of 1.75 (primary outcomes: large for gestational age (LGA), caesarean section, neonatal hypoglycaemia, and cord-blood C-peptide > 90th centile) [4]. The United States (US) National Institutes of Health (NIH) consensus development conference reported that there was insufficient evidence to support the IADPSG recommendations in the absence of a randomised controlled trial (RCT) evaluating clinically important outcomes [5]. However, the World Health Organization (WHO) endorsed IADPSG recommendations, despite noting that the quality of evidence was low and the strength of the recommendation weak [6]. In 2014, ADIPS also endorsed IADPSG recommendations, replacing the ADIPS1998 diagnostic criteria with the IADPSG diagnostic criteria, also known as the ADIPS2014 criteria [7].

Nearly 90% of maternity providers in Australia adopted the IADPSG criteria [8], despite the lack of treatment studies to determine their benefit in terms of pregnancy outcomes. This change in criteria contributed to an almost tripling in incidence of GDM, from 5% in 2007 [9] to 14% in 2019 [10], which posed a major challenge for workforce capacity in managing GDM across Australia.

Studies from the United States (US) and New Zealand have fuelled, rather than resolved this controversy. In the New Zealand GEMS trial, Crowther et al. compared pregnancy outcomes for lower IADPSG glycaemic diagnostic criteria (fasting glucose ≥ 5.1 mmol/L (≥ 91 mg/dL), and/or 1-hour ≥ 10.0 mmol/L (≥ 180 mg/dL), and/or 2-hour ≥ 8.5 mmol/L (≥ 153 mg/dL)) with New Zealand’s former higher glycaemic criteria (fasting glucose ≥ 5.5 mmol/L (≥ 99 mg/dL) and/or 2-hour ≥ 9.0 mmol/L (≥ 162 mg/dL)) and found no difference in LGA risk across the whole obstetric population [11]. However, the study included a subset of 373 women whose OGTT results “fell between” these criteria (fasting glucose 5.1–5.4 mmol/L (91–97 mg/dL) or 2-hour glucose 8.5–8.9 mmol/L (153–160 mg/dL)). For this subset of women, treatment of GDM resulted in better maternal and perinatal outcomes, including less LGA, compared with no treatment [11, 12]. In a US RCT, Hillier et al. compared the one-step new IADPSG diagnostic process with their established 2-step process using the Carpenter and Coustan criteria (fasting glucose ≥ 5.3 mmol/L (≥ 95 mg/dL), and/or 1-hour ≥ 9.9 mmol/L (≥ 178 mg/dL), and/or 2-hour ≥ 8.6 mmol/L (≥ 155 mg/dL) and/or 3-hour ≥ 7.8 mmol/L (≥ 140 mg/dL)). Despite a near doubling in the prevalence of GDM with the IADPSG criteria (16.5% vs. 8.5%), there were no significant differences between groups in any of the primary maternal or perinatal outcomes across the whole obstetric population [13]. Study design prevented a substudy of women whose OGTT results fell between these criteria. Both studies have limitations and have been subject to criticism [14–16].

In other published work, treatment of GDM has been demonstrated to reduce birth centiles across the cohort, which can increase the risk of small for gestational age (SGA); SGA is associated with higher rates of adverse outcomes [17].

During 2016–2017, six neighbouring hospitals in our multiethnic region of Greater Western Sydney, Australia, adopted 1-step antenatal screening for GDM using a 75 g OGTT. Hospital and local pathology providers routinely collected fasting, 1-hour and 2-hour blood glucose levels. Three hospitals were early adopters of the IADPSG criteria, while three other hospitals continued to use the ADIPS1998 criteria. This contemporaneous use of the two sets of criteria at neighbouring hospitals during that time, together with the routine collection of three-point 75 g OGTTs, provided us with a unique opportunity to compare the effect of GDM treatment versus no treatment on pregnancy outcomes for women whose OGTT results fell between these criteria. In our study, we referred to these women as having ‘mild’ GDM, which we defined as GDM diagnosed by either the IADPSG criteria or ADIPS1998 criteria, but not both (fasting glucose 5.1–5.4 mmol/L (91–97 mg/dL) and/or 1-hour glucose ≥ 10.0 mmol/L (≥ 180 mg/dL), or 2-hour glucose 8.0-8.4 mmol/L (144–151 mg/dL)).

The purpose of our study was to investigate the effect of GDM treatment, compared with no treatment, on pregnancy outcomes for women with mild GDM.

## Methods

We conducted a multicentre, retrospective cohort study of 17,512 pregnant women who delivered their baby between 1 January 2016 and 31 December 2017 at one of six neighbouring hospitals in Greater Western Sydney, Australia. Demographic and obstetric data were obtained from hospital obstetric databases (eMaternity, ObstetriX, Mother-Baby Maternal Data Collection). Three-point antenatal 75 g OGTTs were obtained from hospital pathology or local private pathology providers and linked with the obstetric data. Women with pre-existing diabetes or multifetal pregnancies were excluded. If women had more than one OGTT during pregnancy, the most recent one was used. Pregnancy outcomes included LGA, SGA, induction of labour (IOL), caesarean section, gestational hypertension, and preeclampsia. This study was reviewed and approved by Western Sydney Local Health District Human Research Ethics Committee (HREC reference LNR/17/WMEAD/287) and Research Governance Office, South Western Sydney Local Health District Research Governance Office, and Nepean Blue Mountains Local Health District Research Governance Office, and was therefore performed in accordance with the ethical standards of the Declaration of Helsinki in 1995 (as revised in Fortaleza, Brazil, October 2013). Western Sydney Local Health District Research Ethics Committee waived the need for informed consent due to retrospective study.

The presence of GDM by one, both or neither set(s) of diagnostic criteria was evaluated for the entire cohort. For women with ‘mild’ GDM, the effect of treatment versus no treatment on pregnancy outcomes was determined for:

1. Women with IADPSG-only GDM (i.e. fasting glucose 5.1–5.4 mmol/L (91–97 mg/dL) and/or 1-hour glucose ≥ 10.0 mmol/L (≥ 180 mg/dL), and 2-hour glucose < 8.0 mmol/L (< 144 mg/dL)); and.
2. Women with ADIPS1998-only GDM (i.e. fasting glucose < 5.1 mmol/L (< 91 mg/dL) and 1-hour glucose < 10.0 mmol/L (< 180 mg/dL) and 2-hour glucose 8.0-8.4 mmol/L (144–151 mg/dL)).

For both analyses, women who did not have GDM by either set of criteria served as a reference group.

GDM treatment was consistent across all six hospitals, including dietary and lifestyle advice, self-monitoring of blood glucose (SMBG) and insulin if required. Women were advised to perform SMBG four times a day at predetermined intervals (fasting and 2 h after the start of each of three main meals). Glycaemic targets for treatment of women with IADPSG-only GDM were < 5.3 mmol/L and < 7.0 mmol/L for fasting and 2-hour post-prandial glucose levels, respectively. Glycaemic targets for treatment of women with ADIPS1998-only GDM were < 5.5 mmol/L and < 7.0 mmol/L for fasting and 2-hour post-prandial glucose levels, respectively. SMBG records were reviewed at each clinic appointment and insulin therapy was commenced if SMBG levels were above glycaemic treatment targets without dietary or lifestyle explanation. Isophane insulin, an intermediate-acting insulin, was prescribed at bedtime to target elevated fasting SMBG. Either insulin lispro or insulin aspart, which are rapid-acting insulin analogs, was prescribed at the start of a main meal to target the corresponding elevated post-prandial SMBG.

The a priori primary outcome for analysis was the dichotomous variable, LGA (> 90th centile). Secondary outcomes included the dichotomous variables, SGA (< 10th centile) [18], IOL, caesarean section, gestational hypertension, preeclampsia and stillbirth, as well as the continuous variables, gestational age at delivery, birthweight and birth centile. Birth centiles were calculated using the validated customised gestation.net bulk centile calculator Australian version 6.7.3 [19] which adjusts for maternal weight and height, parity, ethnicity, neonatal gender, gestation and birthweight.

### Statistical analyses

All statistical analyses were undertaken by a statistician (KB). IBM SPSS Statistics version 26 was used to analyse the data. Continuous variables were summarised using the mean ± standard deviation (SD). Frequencies and percentages (%) were used for categorical variables. Two-tailed tests with a significance level of 5% were used throughout. Comparison of baseline characteristics between the groups was performed using analysis of variance for continuous variables and chi-squared tests for categorical variables.

Since observations were clustered by hospital, the intra-cluster correlation coefficient was computed for each outcome of interest and used to adjust reported effects. Generalised linear models with a log link function and robust covariance estimates were used to test for interaction between the effects of hospital and GDM group on each of the dichotomous outcome variables after adjusting for maternal age, maternal body mass index (BMI), Socio-Economic Indexes for Areas (SEIFA) score, primiparous status, smoking status and country of birth, and correcting for intra-cluster (hospital) correlation. For each outcome, the model provided an estimate with 95% confidence interval (CI) of the ratio of the relative risk (RR) of the outcome in the GDM group of interest versus women who did not have GDM by either criteria for those who were treated versus those untreated.

Similar adjusted models with a linear link function were used to test for interaction between the effects of hospital and GDM group on each of the continuous outcome variables. Here the model provided an estimate with 95% CI of the change in the difference between the GDM group of interest versus women who did not have GDM by either criteria for those who were treated versus those untreated. Birth centile analyses were not adjusted for maternal country of birth, BMI and age as these variables are already factored into the gestation.net birth centile calculator.

## Results

### Study population

Of 17,512 pregnant women, 12,858 (73.4%) women did not have GDM by either criteria, 2,334 (13.3%) women had GDM by both sets of criteria and 2,320 (13.2%) women had GDM by only one set of criteria (i.e. ‘mild’ GDM).

Of the 2,320 women with ‘mild’ GDM, 1,881 (10.7%) women had IADPSG-only GDM (i.e. fasting glucose 5.1–5.4 mmol/L (91–97 mg/dL) and/or 1-hour glucose ≥ 10.0 mmol/L (≥ 180 mg/dL)), and 439 (2.5%) women had ADIPS1998-only GDM (i.e. 2-hour glucose 8.0-8.4 mmol/L (144–151 mg/dL)). The characteristics of the women are presented in Tables 1 and 2.

**Table 1:** Characteristics of pregnant women in the study with IADPSG-only GDM

	Treated (*n* = 1234)	Untreated (*n* = 647)	*P*-value
Age (years) ± SD	29.7 ± 5.4	30.9 ± 4.9	< 0.001
BMI (kg/m^2^) ± SD	30.2 ± 6.9	28.7 ± 6.8	< 0.001
Parity ± SD	1.2 ± 1.4	1.0 ± 1.3	0.023
SEIFA^†^ Score ± SD	952 ± 58	991 ± 76	< 0.001
Smoking	65 (5.8%)	55 (8.6%)	0.030
Primiparous	468 (37.9%)	254 (39.3%)	0.253
Born in Australia	556 (45.1%)	250 (38.6%)	0.008
Predominant Ethnicity			< 0.001
European	681 (55.2%)	290 (44.8%)	
East Asian	126 (10.2%)	65 (10.0%)	
Indian Subcontinent	144 (11.7%)	201 (31.1%)	
Other	283 (22.9%)	91 (14.1%)	
Gestation of OGTT (weeks) ± SD	23.5 ± 7.0	26.7 ± 3.3	< 0.001

^†^SEIFA – Socio-Economic Indexes for Areas

**Table 2:** Characteristics of pregnant women in the study with ADIPS1998-only GDM

	Treated (*n* = 334)	Untreated (*n* = 105)	*P*-value
Age (years) ± SD	31.3 ± 5.0	29.7 ± 5.5	0.003
BMI (kg/m^2^) ± SD	25.6 ± 5.7	27.4 ± 5.8	0.002
Parity ± SD	0.8 ± 1.0	0.9 ± 1.4	0.09
SEIFA^†^ Score ± SD	991 ± 76	952 ± 58	< 0.001
Smoking	13 (3.9%)	6 (6.1%)	0.401
Primiparous	149 (44.6%)	56 (53.3%)	0.145
Born in Australia	98 (29.3%)	40 (38.1%)	0.117
Predominant Ethnicity			0.020
European	107 (32.0%)	46 (43.8%)	
East Asian	83 (24.9%)	12 (11.4%)	
Indian Subcontinent	101 (30.2%)	12 (11.4%)	
Other	43 (12.9%)	17 (16.2%)	
Gestation of OGTT (weeks) ± SD	25.4 ± 4.4	25.9 ± 3.6	0.14

^†^SEIFA – Socio-Economic Indexes for Areas

### Effect of treatment versus no treatment on pregnancy outcomes for women with IADPSG-only GDM

Women with IADPSG-only ‘mild’ GDM (i.e. fasting glucose 5.1–5.4 mmol/L (91–97 mg/dL) and/or 1-hour glucose ≥ 10.0 mmol/L (≥ 180 mg/dL)) who were treated for GDM were less likely to have a LGA infant (12.8% vs. 16.7%, ratio of RR 0.66, 95%CI 0.49–0.88, *p* = 0.004) and more likely to have IOL (40.4% vs. 30.3%, ratio of RR 1.55, 95%CI 1.03–2.34, *p* = 0.032) than those who were untreated (Tables 3 and 4). Their infants had a lower mean birth centile (52.7 ± 29.1 vs. 54.9 ± 29.9, mean difference − 4.52%, 95%CI -7.96 to -1.08%, *p* = 0.009).

**Table 3:** Effect of treatment on pregnancy outcomes for women with IADPSG-only GDM: categorical outcomes

	Treated (*n* = 1234)	Untreated (*n* = 647)	Adjusted^†^ RR ratio (95% CI)	*P*-value
LGA	149/1165 (12.8%)	108 (16.7%)	0.66 (0.49–0.88)	0.004
SGA	96/1165 (8.2%)	49 (7.6%)	1.26 (0.65–2.44)	0.490
Induction of labour	499 (40.4%)	196 (30.3%)	1.55 (1.03–2.34)	0.032
Caesarean section	364 (29.5%)	227 (35.1%)	0.98 (0.40–2.35)	0.957
Gestational hypertension	47 (3.8%)	25 (3.9%)	0.99 (0.26–3.78)	0.988
Pre-eclampsia	15 (1.2%)	17 (2.6%)	0.76 (0.16–3.58)	0.726

^†^Adjusted for maternal age, maternal BMI, SEIFA score, primiparity, smoking, and whether born in Australia and corrected for clustering

LGA – large for gestational age; SGA – small for gestational age

**Table 4:** Effect of treatment on pregnancy outcomes for women with IADPSG-only GDM: continuous outcomes

	Treated (*n* = 1234)	Untreated (*n* = 647)	Adjusted^†^ change in difference (95% CI)	*P*-value
Gestational age (weeks)	38.7 ± 1.6	39.1 ± 1.5	-0.11 (-0.86, 0.64)	0.767
Birthweight (g)	3377 ± 504	3439 ± 546	-88.7 (-313.5, -136.0)	0.430
Birth centile	52.7 ± 29.1	54.9 ± 29.9	-4.52 (-7.96, -1.08)	0.009

^†^Adjusted for maternal age, maternal BMI, SEIFA score, primiparity, smoking, and whether born in Australia and corrected for clustering

All values are mean ± SD unless otherwise stated

### Effect of treatment versus no treatment on pregnancy outcomes for women with ADIPS1998-only GDM

For women with ADIPS1998-only ‘mild’ GDM (i.e. 2-hour glucose 8.0-8.4 mmol/L (144–151 mg/dL)), there were no significant differences in pregnancy outcomes between those who were treated for GDM and those who were untreated (Tables 5 and 6).

**Table 5:** Effect of GDM treatment on pregnancy outcomes for women with ADIPS1998-only GDM: categorical outcomes

	Treated (*n* = 334)	Untreated (*n* = 105)	Adjusted^†^ RR ratio (95% CI)	*P*-value
LGA	28 (8.4%)	10 (9.5%)	1.00 (0.46–2.20)	0.992
SGA	38 (11.4%)	12 (11.4%)	0.89 (0.28–2.81)	0.834
Induction of labour	121 (36.2%)	26 (24.8%)	1.36 (0.50–3.69)	0.537
Caesarean section	110 (32.9%)	35 (33.3%)	0.85 (0.15–4.96)	0.853
Gestational hypertension	5 (1.5%)	2 (1.9%)	0.86 (0.02-46)	0.940
Pre-eclampsia	5 (1.5%)	0 (0%)	^‡^	

^†^Adjusted for maternal age, maternal BMI, SEIFA score, primiparity, smoking, and whether born in Australia and corrected for clustering

^‡^RR cannot be calculated when there are no cases in one group

LGA – large for gestational age; SGA – small for gestational age

**Table 6:** Effect of GDM treatment on pregnancy outcomes for women with ADIPS1998-only GDM: continuous outcomes

	Treated (*n* = 334)	Untreated (*n* = 105)	Adjusted^†^ change in difference (95% CI)	*P*-value
Gestational age (weeks)	39.0 ± 1.7	38.9 ± 1.8	-0.24 (-2.05, 1.57	0.791
Birthweight (g)	3229 ± 534	3349 ± 519	-106.3 (-587, 374)	0.658
Birth centile	47.7 ± 29.6	53.2 ± 28.3	-3.19 (-10.4, 3.99)	0.374

^†^Adjusted for maternal age, maternal BMI, SEIFA score, primiparity, smoking, and whether born in Australia and corrected for clustering

All values are mean ± SD unless otherwise stated

## Discussion

In this large Australian multicentre study, 13.2% pregnant women had ‘mild’ GDM, depending on whether the IADPSG criteria or ADIPS1998 criteria were used. Women who were treated according to IADPSG-only GDM criteria (i.e. fasting glucose 5.1–5.4 mmol/L (91–97 mg/dL) and/or 1-hour glucose ≥ 10.0 mmol/L (≥ 180 mg/dL), and 2-hour glucose < 8.0 mmol/L (< 144 mg/dL)) had lower rates of LGA infants and lower birth centile infants than untreated women. They had higher rates of induction, but no increase in caesarean section rates. Women who were treated according to ADIPS1998-only GDM criteria (i.e. fasting glucose < 5.1 mmol/L (< 91 mg/dL) and 1-hour glucose < 10.0 mmol/L (< 180 mg/dL) and 2-hour glucose 8.0-8.4 mmol/L (144–151 mg/dL)) had no differences in pregnancy outcomes compared with untreated women.

The two major GDM treatment studies, the Australian Carbohydrate Intolerance Study in Pregnant Women (ACHOIS) in 2005 and Landon et al. in 2009, demonstrated that treatment of GDM improves perinatal outcomes, including the rate of macrosomia [20, 21]. However, ACHOIS recruited women with a 2-hour glucose 7.8–11.0 mmol/L (140–198 mg/dL) but fasting glucose < 7.8 mmol/L (< 140 mg/dL), and Landon et al. included women with 2 elevated post-prandial OGTT levels using the Carpenter and Coustan criteria but fasting glucose < 5.3 mmol/L (< 95 mg/dL), so the findings of these studies cannot be extrapolated to women diagnosed by the IADPSG criteria.

Key concerns regarding adoption of the IADPSG criteria in Australia centred on the lack of RCT evidence of benefit in treating GDM diagnosed by these criteria, particularly with the lower fasting glucose threshold, as well as the potential overdiagnosis of GDM [22–25]. A counterargument is that the IADPSG criteria are based on glucose thresholds associated with diabetic fetopathy, whereas the ADIPS1998 criteria are less evidence-based [26, 27].

The US and New Zealand RCTs added to the controversy. The US study by Hillier et al. did not find a difference in outcomes at a population level using the IADPSG criteria compared with the original two-step approach using Carpenter and Coustan criteria [13]. However, women with GDM were a relatively small proportion of the total obstetric population, so signals for potential benefit in a subset of women with GDM may have been diluted. Similarly, the New Zealand GEMS study did not show a difference in LGA rate at a population level using the IADPSG criteria compared with their old criteria (which are similar to the ADIPS1998 criteria) [11]. However, this study may have been underpowered to demonstrate whether there was a population effect, and the rates of GDM overall in the recruited population were smaller than many centres report, so the study may not be representative of other populations. Despite these limitations, they identified a subset of women whose OGTT results “fell between” the old and new criteria who may benefit from treatment, resulting in fewer LGA infants. Our results are consistent with this finding.

Our observational cohort study provides much needed Australian pregnancy outcome data to inform the debate on diagnostic criteria for GDM. The finding that women with IADPSG-only GDM who were treated for GDM had fewer LGA infants is consistent with the wider evidence that treatment of GDM reduces the rate of LGA or macrosomia [11, 28–33]. Our findings are also in keeping with the study by Meek et al. [34] comparing the IADPSG criteria with the National Institute for Clinical Excellence (NICE) criteria (which are similar to the ADIPS1998 criteria). In that study, untreated women who were diagnosed with GDM according to the IADPSG criteria but not the NICE criteria had a higher risk of having an LGA infant and caesarean delivery.

It is notable that, despite a reduction in LGA in our study for women with IADPSG-only GDM who were treated for GDM, there was an increased rate of IOL. This finding is consistent with other studies demonstrating that women treated for GDM are more likely to experience IOL [28]. IOL is commonly performed to reduce the risk of late pregnancy complications, including stillbirth and shoulder dystocia [35, 36].

Importantly, we also found that treatment of women with ADIPS1998-only GDM did not significantly change pregnancy outcomes. While numbers were small, this is a novel finding that supports the higher IADPSG 2-hour diagnostic threshold.

A strength of our study is that the cohort included all pregnant women with available OGTT results, not just women who had been given a GDM diagnosis. Our study design enabled us to analyse data for women within any category of glycaemia. We were therefore able to identify all pregnant women with ‘mild’ GDM, irrespective of whether they received a clinical diagnosis of GDM or not. Further, the fact that women at some hospitals were diagnosed and managed for GDM, while women at neighbouring hospitals were not, enabled us to compare the effect of treatment versus no treatment on pregnancy outcomes. In the absence of a multiethnic Australian RCT, this large, multiethnic, multicentre cohort study with a ‘normal’ comparison group, is arguably the best alternative.

A limitation of our study is that although the women were from neighbouring hospitals, there were significant differences in demographic factors between the subgroups, but we did adjust for these differences using statistical methods. It is possible that there were other unidentified differences in inter-hospital practice, such as indication for IOL, that may limit the interpretability of our results. Further, other relevant outcomes were unable to be measured. For example, it would be of interest to know if the untreated IADPSG-only GDM women also had higher rates of neonatal hypoglycaemia. However, as these women were not diagnosed with GDM in their clinical setting, their infants were not routinely screened for hypoglycaemia. This is an important issue when considering different diagnostic criteria. Another limitation of our retrospective study is the reliance on electronic datasets. At our hospitals, treatment modality, maternal glucose levels following diagnosis, and neonatal length are not routinely collected in our obstetric databases, and are acknowledged as potential confounders. Finally, the fasting glycaemic targets for treatment were different between the IADPSG-only GDM group (< 5.3 mmol/L) and ADIPS1998-only GDM group (< 5.5 mmol/L). However, our study did not compare these two groups. Instead, we investigated the effect of treatment versus no treatment in each of these two separate cohorts. Therefore, the results of our study cannot be attributed to different treatment targets.

In summary, the findings of our study suggest that for the subset of women with a fasting glucose 5.1–5.4 mmol/L (91–97 mg/dL) and/or 1-hour glucose ≥ 10.0 mmol/L (≥ 180 mg/dL) (but 2-hour glucose < 8.0 mmol/L (< 144 mg/dL)) on their antenatal OGTT, treating GDM to glycaemic targets of < 5.3 mmol/L fasting and < 7.0 mmol/L 2-hour post-prandial results in less LGA, but more IOL, compared to not treating. Further, treating women whose GDM diagnosis is based on an isolated 2-hour glucose 8.0-8.4 mmol/L (144–151 mg/dL) may not be beneficial. The results of our study highlight the importance of treating even mild forms of IADPSG-GDM to improve clinically significant pregnancy outcomes.
